# The necessity of ulnar osteotomy in children with missed Monteggia fractures

**DOI:** 10.3389/fped.2025.1636165

**Published:** 2025-11-20

**Authors:** Man Zhang, Yu Zhou, Peikang Wang, Xinkai Zhang, Hailun Yao, Di Li, Xing Liu

**Affiliations:** Department of Orthopedic, Children’s Hospital of Chongqing Medical University, Chongqing, China

**Keywords:** missed Monteggia fracture, child, osteotomy, open reduction, Kirschner wire fixation

## Abstract

**Objective:**

The aims of this study were to review our cases of missed Monteggia fracture treated by open reduction of the radial head with or without ulnar osteotomy and to investigate the necessity of ulnar osteotomy in the surgical treatment of a missed Monteggia fracture.

**Materials and methods:**

The children were divided into two groups according to the surgical method used. The patients in group A were treated with Kirschner wire or external fixation after ulnar osteotomy and Kirschner wire fixation after open reduction of the humeroradial joint. The patients in group B were treated with open reduction of the humeroradial joint and Kirschner wire fixation and ulnar osteotomy was not performed. The imaging results from the most recent postoperative follow-up were evaluated using the Nakamura grading criteria, and the patients’ elbow function was evaluated using the Mayo Elbow Performance Score (MEPS).

**Results:**

There were 53 patients in group A and 14 patients in group B. The mean age of the children in group A was older than that in group B and the time to diagnosis and treatment in group A was longer than that in group B. Regarding the Nakamura grading, there was no significant difference in the proportion of those that received an “excellent” postoperative score between group A and group B (*p* > 0.05). There was no significant difference in the MEPS post-operation between group A and group B (*P* > 0.05). In group A, the recorded complications included a postoperative dislocation of the humeroradial joint, a broken Kirschner wire in the humeroradial joint, occasional mild pain in the elbow joint, and Kirschner wire ends penetrating the patient’s subcutaneous tissue, all of which were treated through surgery. In group B, the recorded complications were a postoperative dislocation of the humeroradial joint, a broken Kirschner wire in the humeroradial joint, and myositis ossificans.

**Conclusion:**

Ulnar osteotomy is not necessary for all children with missed Monteggia fractures. Open reduction of the humeroradial joint followed by Kirschner wire fixation is a surgical option in younger patients with a short time to diagnosis and treatment .

## 1 Introduction

The Monteggia fracture-dislocation, described by Giovanni Battista Monteggia in 1814, is defined as an ulnar fracture associated with dissociation of the proximal radioulnar joint and dislocation of the radial head (1). A Monteggia-fracture dislocation is a relatively rare injury, accounting for approximately 1% of all pediatric forearm fractures (2). There is no uniform definition of the interval required after a trauma for a diagnosis of a chronic Monteggia fracture. Some authors argue that a lesion should be defined as chronic when the radial head dislocation or subluxation has persisted for more than 4 weeks (3).

A detailed anatomical analysis of Monteggia fractures was conducted by Ring et al. (4). In a Monteggia fracture, the annular and quadrate ligaments are ruptured, allowing for a dissociation of the proximal radioulnar joint and radiocapitellar articulation due to associated elbow capsular disruptions, but the majority of the interosseous membrane and the triangular fibrocartilage complex at the distal radioulnar joint remain intact.

Clinically, a wide range of symptoms may be present, including pain, mobility limitation (mainly during flexion and pronation) (5), progressive valgus deformity, filling of the space originally occupied by the dislocated radial head (6), lateral elbow instability, and even late ulnar nerve paralysis (7).

Correction of the ulnar deformity through osteotomy is the most important factor in the reduction and consequent preservation of the radial head (3). However, osteotomy causes significant trauma in patients. Thus, an early diagnosis and adequate treatment of Monteggia fractures result in excellent outcomes, and allow for conservative management in most cases, without complex surgical procedures involving ulnar osteotomies. Whether ulnar osteotomy is necessary in all children with early missed Monteggia fractures remains controversial. Some scholars have reported satisfactory results with isolated open reduction of the humeroradial joint in children with missed Monteggia fractures (8, 9). Park et al. (10) also proposed indications for open reduction without ulnar osteotomy. Our study reviewed cases of missed Monteggia fracture treated by open reduction of the radial head with or without ulnar osteotomy in our hospital and investigated the necessity of ulnar osteotomy in the surgical treatment of a missed Monteggia fracture.

## 2 Materials and methods

The study was conducted at a tertiary care hospital in Chongqing, China. Informed consent was obtained from the parents of the children in our study. The study was conducted according to the principles outlined in the Declaration of Helsinki. This study was approved by the Medical Research Ethics Committee of our hospital (Approval No. 2024-058) on 20 January 2024. In this study, a retrospective analysis was performed on children who were diagnosed with a missed Monteggia fracture in our hospital from July 2014 to October 2023 and who received surgical treatment. All the operating surgeons were qualified to perform the procedure and held the professional title of deputy chief physician or higher. Prior to the initiation of any surgery in this study, the surgical approach was collectively determined through discussions among all the physicians within the department. After excluding children with congenital dislocation of the radial head and those who had received surgical treatment in other hospitals after their injury, a total of 67 children were included, all of whom had more than 3 weeks from injury to surgery.

The children were divided into two groups according to the surgical method used. The children in group A, aged 1–15 years, were treated with Kirschner wire or external fixation after ulnar osteotomy using a subcutaneous border approach to the ulna and Kirschner wire fixation after humeroradial joint open reduction using the Kocher approach. In group B, the age of children ranged from 2 to 9 years, only open reduction of the humeroradial join using the Kocher approach was conducted, and the joint was fixed with Kirschner wires. Ulnar osteotomy was not performed in these patients. The imaging results from the most recent postoperative follow-up were evaluated using the Nakamura grading criteria, and the elbow function was evaluated using the Mayo Elbow Performance Score (MEPS; 90–100 = excellent, 75–89 = good, 60–74 = fair, ≤60 poor). The redislocation rate of the humeroradial joint in each group was also recorded.

The data were recorded and managed in Microsoft Excel. All the statistical analyses were performed using IBM SPSS Statistics, version 26.0. Continuous variables with normal distribution are presented as mean ± standard deviation (SD), and groups were compared using *t*-tests. Non-normally distributed data are presented as median (interquartile range, IQR) and were compared using the rank-sum test. Categorical variables are expressed as number (*n*) and percentage (%) and were compared using the chi-square test or Fisher's exact test. A *p*-value <0.05 was considered statistically significant.

## 3 Result

### 3.1 Comparison of general clinical data

There were 53 patients in group A, including 32 boys and 21 girls, with 21 left-sided injuries and 32 right-sided injuries. The mean age of these patients at operation was 7.3 years old (1–15 years old), the median time to diagnosis and treatment was 5.0 months (0.8–108 months), and the mean follow-up time was 12.5 months (2–54 months). Regarding the Bado classification of these cases, 35 were type I and 18 were type III.

There were 14 patients in group B, including 3 boys and 11 girls, with 6 left-sided and 8 right-sided injuries. The mean age of these patients at operation was 5.1 years old (2–9 years old), the median time to diagnosis and treatment was 1.35 months (0.7–6 months), and the mean follow-up time was 15.5 months (5–36 months). Regarding the Bado classification of these cases, 11 were type I and 3 were type III.

### 3.2 Comparison of Nakamura grade, Mayo grade, and postoperative redislocation rate between group A and group B

The age of patients in group A and group B at the time of operation was compared using the independent sample *t*-test, and the rank-sum test was used to compare the time to diagnosis and treatment between the two groups. Fisher’s exact test was used to compare the postoperative “excellent” Nakamura imaging grade, “excellent” postoperative MEPS, and dislocation rates between the two groups. The results are shown in Table 1.

**Table 1:** Comparison of Nakamura grade, Mayo grade, and dislocation rate between group A and group B.

Title	Group A	Group B	*t*/Z/χ^2^	*P*
Mean age (years) (X ± SD)	7.3 ± 3.0	5.1 ± 2.1	2.566	0.013
Median time to diagnosis and treatment in months [M(P_25_,P_75_)]	5.0 (1.9, 24.0)	1.35 (1.0, 3.5)	−3.099	0.002
Nakamura grade: excellent	90.6%	92.9%	0.000	1.000
Mayo Elbow Performance Score: excellent	84.6%	92.9%	0.247	0.619
Dislocation rate	7.5%	7.1%	0.000	1.000

The mean age of the children in group A was older than that in group B (*P* = 0.031), and the time to diagnosis and treatment in group A was longer than that in group B (*P* = 0.002).

There was no significant difference in the proportion of excellent Nakamura grades between groups A and B (*P* = 1.000). There was no significant difference in MEPS after the operation between groups A and B (*P* = 0.619). There was no significant difference in the redislocation rate between the two groups (*P* = 1.000).

### 3.3 Case presentation

**Figure 1:**
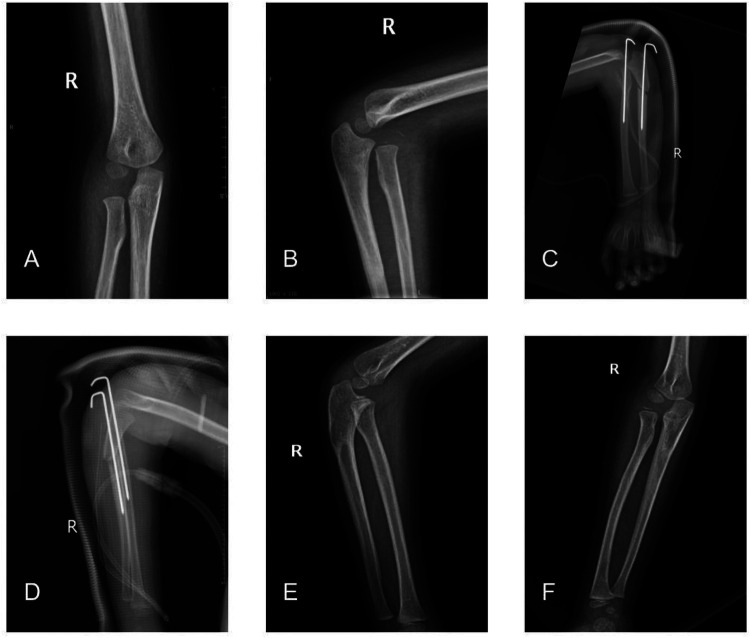
**(A,B)** Preoperative anteroposterior and lateral images of the right elbow, with an anterior dislocation of the right radial head. **(C,D)** Images of the ulnar osteotomy with Kirschner wire internal fixation combined with Kirschner wire internal fixation of the humeroradial joint on the first day after surgery. **(E,F)** Anteroposterior and lateral images of the right elbow at the 1-year postoperative follow-up, with good healing at the ulnar osteotomy site and good alignment of the right humeroradial joint.

The first patient presented here is from group A. The 4-year-old male patient presented with a right elbow mobility disorder 3 months after injury and locally palpable bony protrusions accompanied by tenderness, and was diagnosed with a Bado type I missed Monteggia fracture (Figure 1).

**Figure 2:**
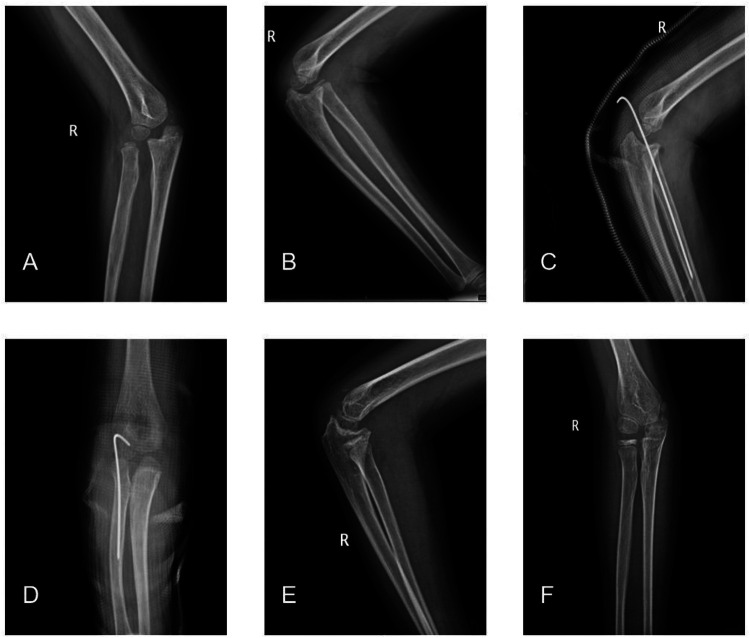
**(A,B)** Preoperative anteroposterior and lateral images of the right elbow showing an anterior lateral dislocation of the right radial head. **(C,D)** Anterolateral images of the right elbow on the first day after the open reduction and internal Kirschner wire fixation of the humeroradial joint. **(E,F)** A 2-year postoperative follow-up x-ray of the right elbow joint, with good alignment of the right humeroradial joint.

The second patient presented here is from group B. The 5-year-old male patient was diagnosed with a Bado III missed Monteggia fracture and suffered from a right elbow mobility disorder and local tenderness 5 months after injury (Figure 2).

## 4 Complications

After the operation, two cases in group A had a broken Kirschner wire in the humeroradial joint, one of which was accompanied by a dislocation of the humeroradial joint. The children occasionally experienced a mild, prickling pain, typically induced by writing or changes in the weather, which resolved spontaneously without the need for intervention. Redislocations of the humeroradial joint were found in three cases 3 months after operation. In one case, the end of the Kirschner wire in the humeroradial joint had migrated into the skin more than 1 month after the operation, and the Kirschner wire was removed through surgery in hospital.

In group B, a postoperative dislocation of the humeroradial joint, a broken Kirschner wire in the humeroradial joint, and myositis ossificans occurred in one case, respectively.

## 5 Discussion

Ulnar osteotomy is the key procedure in achieving and maintaining reduction (2, 3), as it addresses the primary deformity of the ulna and aims to restore the normal relationship between the radius and ulna and the width of the interosseous membrane (7). At the same time, the tension of the interosseous membrane stabilizes the position of the radius and prevents the radius head from escaping from the articular sac again after the operation (11). In a study comparing the length of the ulna on the affected and normal sides of children with missed Monteggia fractures, the authors found that the length of the ulna was shorter on the affected side than on the normal side. They argued that a dislocation of the radial head affects the growth of the ulna. Specifically, changes in the axial load from the wrist to the forearm and pressure loss from the head of the humerus to the head of the radius may affect the growth of the ulna, especially during growth spurts, as they found that abnormal differences in the ulna on the affected vs. normal sides were negatively correlated with the age of the injury (12).

There have been studies exploring the necessity of ulnar osteotomy. Park et al. (10), in order to explore the indications for an open reduction of the humeroradial joint instead of ulnar osteotomy, retrospectively analyzed 22 patients with missed Monteggia fractures and measured their maximum ulnar bow (MUB) using lateral radiographs of the forearm. By measuring the maximum distance between the olecranon and the metaphyseal end of the distal ulna to the dorsal border of the ulna and the maximum ulnar arch position, they found that when the MUB was less than 4 mm and the MUB position was 40% of the distal ulna, the reduction of the radius head could be achieved without the need for ulnar osteotomy, suggesting that patients with smaller MUBs and those with an MUB near the distal ulna were unlikely to require ulnar osteotomy.

In our study, the patients in group B were treated with an open reduction of the humeroradial joint and fixed with Kirschner wire, and ulnar osteotomy was not performed. However, there was no statistical difference in the postoperative imaging grades, functional scores, and redislocation rate between groups A and B. Combined with the previous discussion, older age and a longer delay in diagnosis and treatment are risk factors for adverse postoperative imaging and clinical outcomes (13). However, the average age of the children in group B was lower than that in group A, the time to diagnosis and treatment in group B was shorter than that in group A, and the efficacy of the two groups was comparable. Thus, we argue that ulnar osteotomy is not necessary in all children with missed Monteggia fractures. Considering the small sample size of this study, a larger sample study is needed for more exact results.

## 6 Limitations

While a multivariate logistic regression is required to identify independent risk factors for the outcome, our cohort's size was insufficient to support such an analysis. Therefore, it is necessary to expand the sample size for more accurate conclusions. Moreover, because some patients in our study had non-standard forearm x-ray imaging data, incorporating the MUB parameter into the analysis may have introduced bias in the results.

## 7 Conclusion

Ulnar osteotomy is not necessary for all children with missed Monteggia fractures. Open reduction of the humeroradial joint followed by Kirschner wire fixation is a surgical option in younger patients with a short time to diagnosis and treatment.

## Data Availability

The original contributions presented in the study are included in the article/Supplementary Material, further inquiries can be directed to the corresponding author.
